# “In Situ” Methotrexate Injection Followed by Hysteroscopic Resection for Caesarean Scar Pregnancy: A Single-Center Experience

**DOI:** 10.3390/jcm12062304

**Published:** 2023-03-16

**Authors:** Anthony El Sabbagh, Ihsan Sayour, Zaki Sleiman, Gabriele Centini, Lucia Lazzeri, Matteo Giorgi, Errico Zupi, Nassir Habib

**Affiliations:** 1 Department of Surgery, Haykel Hospital, Tripoli 1300, Lebanon; 2 Department of Obstetrics and Gynecology, Lebanese American University Medical Center-Rizk Hospital, Beirut 1100, Lebanon; 3 Department of Molecular and Developmental Medicine, Obstetrics and Gynecological Clinic, University of Siena, 53100 Siena, Italy; 4 Department of Obstetrics and Gynecology Francois Quesnay Hospital, 78201 Mantes-La-Jolie, France

**Keywords:** caesarean scar pregnancy, methotrexate, hysteroscopy, ectopic pregnancy, conservative management

## Abstract

Background: We evaluated the efficacy of local methotrexate (MTX) treatment followed by hysteroscopic resection for caesarean scar pregnancy and its impact on future fertility. Methods: Monocentric, prospective, observational study performed in the Haykel Hospital between June 2016 and December 2020. Twenty-one women with caesarean scar pregnancy underwent a transcutaneous ultrasound-guided direct injection of MTX into the gestational sac in an outpatient setting. Hysteroscopic resection of residual trophoblastic retention was then performed according to perisaccular blood flow. Main results: Two patients had complete spontaneous trophoblast expulsion after MTX injection, and hysteroscopy was performed in 19 patients for residual trophoblastic retention 1 to 12 weeks after MTX injection. Successful preservation of a healthy uterus with the combined procedure was obtained in 94.8% of patients. Hemostatic hysterectomy was required in one patient. Mean hospitalization duration was 1.5 days. Three patients had spontaneous pregnancy after the procedure. Conclusion: Direct MTX injection into the gestational sac for caesarean scar pregnancy followed by hysteroscopic resection was an effective technique with a short hospitalization, fertility preservation and a low major complication rate compared with other modalities of treatment reported in the literature. Further larger prospective comparative studies are needed to confirm the efficacy of this procedure.

## 1. Introduction

A caesarean scar pregnancy (CSP) is a subtype of ectopic pregnancy where the embryo is implanted in the muscle or fibrous tissue of the scar resulting from a previous caesarean section (C-section) [1]. This specific localization was described for the first time in 1978, and the recent literature indicates that CSP is more common than previously assumed, based upon mostly case reports and case series [2,3].

The overall prevalence of ectopic pregnancy is approximately 2% and 6.1% of these I are localized at the level of a previous C-section, with an overall incidence between 0.05 and 0.04% (1:1800–1:2226) [2]. However, in recent decades, its incidence is rising, due to the increasing rate of caesarean deliveries and improved availability and accuracy of early pregnancy ultrasound [4,5].

Ruptured ectopic pregnancy remains the principal specific cause of maternal hemorrhage-related death [6], particularly in the first trimester [7].

According to Rotas et al., an early diagnosis is crucial for fertility preservation, and about half (52%) of CSP occur in those patients with only one caesarean section with a mean gestational age at the time of diagnosis of 7.5 ± 2.5 weeks [8].

Treatment options for CSP include several surgical techniques, such as dilatation and curettage (D&C), laparoscopic or hysteroscopic resection of gestational mass, uterine artery embolization and hysterectomy [9]. Medical options are available to either directly treat the disease or ease the surgical treatment and they generally consist of intravenous or site injection of methotrexate.

International guidelines are well standardized in the administration of methotrexate at a dose of 50 mcgr/m^2^ for tubal localization, with eligibility criteria and a strict follow-up leading to a risk of tubal rupture in 33% of cases within 6 days [10].

Conservative surgical reports of systemic methotrexate administration for non-tubal ectopic pregnancy have shown high failure rates of up to 25% with many patients requiring subsequent surgical intervention [7]. Combined and sequential treatments have been described.

The current literature is mostly based on case reports or case series and there is no consensus regarding optimal treatment. Despite known complications, some patients can elect to continue their pregnancy. However, this is associated with the risk of morbidly adherent placenta and often consequent hysterectomy [11].

The aim of this study is to evaluate the efficacy of local methotrexate injection in scar pregnancy followed by hysteroscopic resection of the residual pregnancy material.

## 2. Materials and Methods

### 2.1. Study Setting, Selection Criteria and Patient Assessement

This prospective, observational, single-center study was conducted in a tertiary care Hospital (Haykel Hospital, Tripoli, Lebanon) between January 2016 and December 2020. All women referred to our center with a diagnosis of caesarean scar pregnancy were assessed for eligibility.

The patients were then enrolled in the study with the following inclusion criteria: CSP demonstrated with transvaginal ultrasound, patient older than 18 years and able to express consent to participate in the study, no major comorbidities, stable hemodynamic condition.

An additional criterion to be included was thickness of the myometrium layer above the gestational sac of 3 mm or more and a negative “sliding sign”, intended as the impossibility to displace the gestational sac from its position using gentle pressure with a transvaginal probe [12].

**Figure 1:**
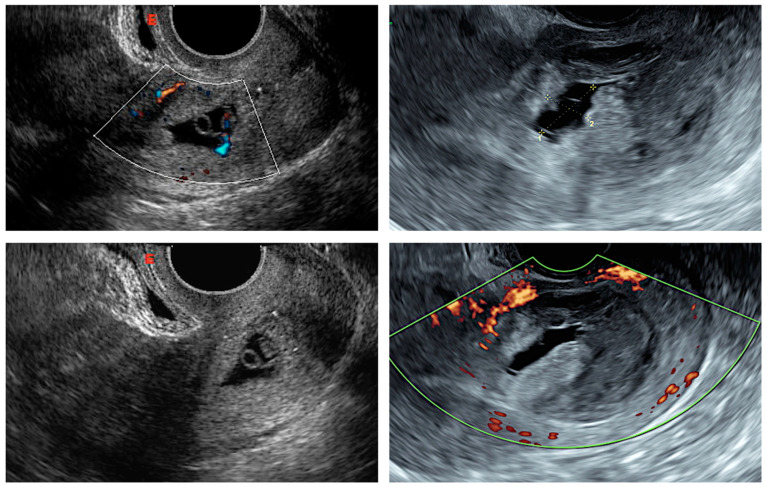
Transvaginal sonographic views of cesarean scar pregnancy.

The diagnosis of CSP was defined on the following transvaginal sonographic criteria [13]:

1. empty uterine cavity with a clearly visible endometrium and empty cervical canal;
2. the presence of a gestational sac, with or without fetal cardiac activity, embedded and surrounded by the myometrium, in the anterior part of the uterine isthmus (Figure 1);
3. peritrophoblastic blood flow surrounding the CSP appearing on Doppler flow sonography.

All patients were prepared in an outpatient setting. Baseline complete blood count, human chorionic gonadotropin dosage, liver and renal function tests and coagulation tests were performed. Gestational age was determined according to the last menstrual period, sac dimension and first trimester crown-to-rump length measurement.

A detailed discussion with the patient was carried out, describing the risks of persistent CSP, including uterine rupture, significant hemorrhage and morbidly adherent placenta. Treatment options were discussed, including local methotrexate injection, surgical treatments (hysteroscopic resection) and the option of continuing the pregnancy.

The risk of treatment failure and possibility to undergo a hysterectomy were explained. Informed consent was signed by every patient before the treatment.

For research purposes, the following anamnestic and clinical data were recorded: age, number of previous C-sections, perisaccular blood flow during transvaginal ultrasound first diagnosis, gestational age at MTX injection, time between MTX injection and hysteroscopic resection, MTX injection and hysteroscopic complications, need for blood transfusion, hospitalization time and final status according to follow-up exams.

### 2.2. Local Methotrexate Injection

The procedure was performed in the fetal–maternal unit in an ambulatory setting, where the patient was placed in the lithotomy position and, after local anesthesia, the injection of 50 mcg of Methotrexate in the gestational sac using a 17-gauge double lumen oocyte pick-up needle was performed, guided by transvaginal ultrasound.

Patients with more than 11 weeks of gestation were initially classified as having a higher risk of treatment failure and hemorrhage, since the available literature reported no hysteroscopic removal of CSP above this gestational week [14,15].

Patients were discharged after the procedure and controlled in an outpatient setting, where the embryo heart activity and the perisaccular blood flow were checked weekly using ultrasound, as a tool for scheduling the hysteroscopic resection for the residual pregnancy product.

For practical purposes, the perilesional blood flow was categorized in 3 classes: weak, enhanced and strong. A weak blood flow corresponded to a color score of 2, according to the IETA blood flow assessment [16]. Enhanced and strong perisaccular vascularization reflected a color score of 3 and 4, respectively. A weakening or a significant reduction in the blood flow in color Doppler was considered a requirement to proceed to hysteroscopic resection.

### 2.3. Hysteroscopic Procedure

Hysteroscopic removal of conceptive tissues was performed by a single experienced operator (E.S.A.) in an inpatient setting using a 26 French (8.5 mm) rigid bipolar continuous-flow resectoscope (Karl Storz GmbH, Tuttlingen, Germany) equipped with a 12° optic. The hysteroscope was introduced in the uterine cavity after dilation of the external os of the cervix with Hegar dilators and transabdominal ultrasound guidance. Normal saline was used for distension and irrigation of the uterine cavity. CSP was removed under direct vision until the myometrium was visualized. Electrical coagulation was used to control bleeding with the use of a wire loop or a rollerball. In cases of postprocedural hemorrhage, uterine tamponade was performed using a Foley catheter inflated with 20 mL of saline solution.

### 2.4. Discharge and Follow-Up

Patients were discharged the same day if they felt well and were clinically stable.

Follow-up included a pelvic exam and a transvaginal ultrasound 30 days and 12 months after the surgical procedure.

### 2.5. Study Outcomes

The primary outcome was the percentage of patients with a complete resolution of the CSP with the combined approach (local injection plus hysteroscopic procedure) and preservation of the uterus.

The secondary outcomes were the percentages of:

- overall and specific surgical complications;
- postprocedural pregnancy;
- perisaccular blood flow.

Time of hospitalization expressed in days was another outcome.

### 2.6. Statistical Analysis

The statistical analyses were performed with SPSS version 28.0 (SPSS Inc, Chicago, IL, USA). Numerical variables were summarized as mean ± standard deviation (SD) or mean and range; categorical variables were summarized as counts and percentages.

## 3. Results

During the study period, a total of 21 patients were consecutively recruited for study. Patients’ clinical details are summarized in Table 1.

The mean (±SD) age at diagnosis of the study sample was 30.3 ± 3.4. Eighteen patients (85.7%) had had more than one previous C-section (mean ± SD: 2.7 ± 2.3).

The assessment of the gestational sac vascularization during the first transvaginal ultrasound demonstrated weak blood flow in nine cases (*n* = 9/21, 42.9%), strong in four cases (*n* = 4/21, 19.0%) and enhanced in eight cases (*n* = 8/21, 38.1%).

Local methotrexate injection in the gestational sac was successfully performed in all cases. Gestational age at injection ranged between 6 and 11 weeks of gestation (mean ± SD: 7.7 ± 1.2). There were no complications during MTX puncture.

Amongst the 21 patients who received the methotrexate injection, 2 patients (9.5%) had complete and spontaneous expulsion of the gestational sac after 2 weeks and 19 patients underwent hysteroscopic resection of the residual trophoblast product after a mean of 5.2 weeks following the injection, ranging from 1 to 12 weeks.

**Figure 2:**
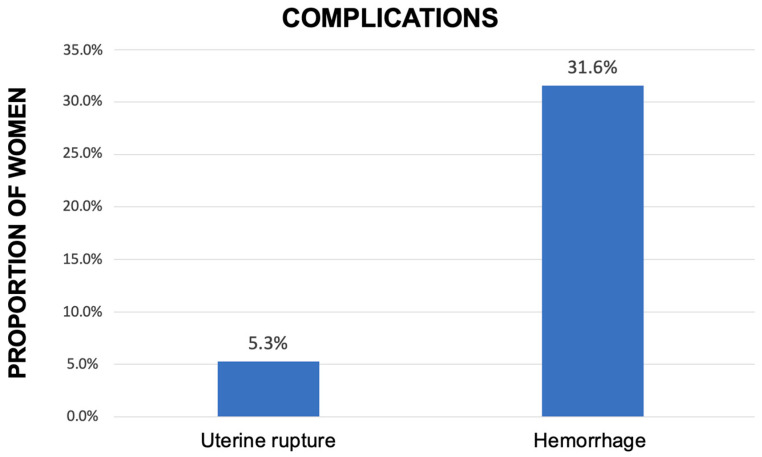
Rate and type of complication among the patients treated with both methotrexate injection and subsequent hysteroscopic resection (expressed in percentage, %).

Overall, local injection plus surgical procedure had a success rate of 94.8% (*n* = 18/19), with preservation of a healthy uterus. One patient underwent emergency hysterectomy due to incontrollable hemorrhage caused by uterine rupture 2 days after the hysteroscopic resection. As shown in Figure 2, the overall complication rate in the group treated with the combined technique was 36.9% (*n* = 7), represented by six (31.6%) postprocedural hemorrhages successfully treated by Foley intrauterine tamponade balloon and one (5.2%) emergency hysterectomy due to uterine rupture with massive bleeding. Packed red blood cell transfusion of 1 unit for a bleeding < 1000 mL was required in three patients (*n* = 3/19, 15.8%).

**Figure 3:**
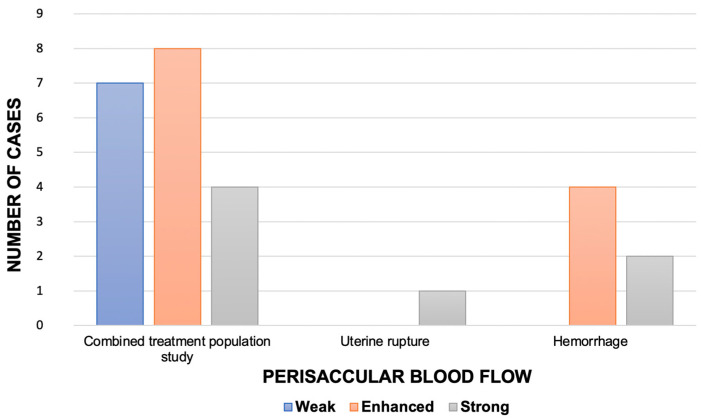
Perisaccular blood flow detected using color Doppler at first scan (expressed as number of cases, *n*) in all the patients treated with the combined approach (methotrexate injection plus hysteroscopic resection) and according to the reported complication.

As reported in Figure 3, strong blood flow during the first transvaginal ultrasound was detected in 4 (21.1%) of the 19 patients treated with local injection of MTX and hysteroscopy. In the same group, enhanced and weak blood flow were reported in eight cases (42.1%) and seven cases (36.8%), respectively. Among the hemorrhage group, strong vascularization was detected in two patients (*n* = 2/6, 33.3%); remaining cases of the same group were all characterized by enhanced blood flow (*n* = 4/6, 66.6%). Weak blood flow was solely associated with a safe procedure without complications.

The only case of uterine rupture had a strong color Doppler at the first ultrasound scan.

Among those successfully treated, 15.8% (*n* = 3/19) conceived and carried out a successful later pregnancy delivering at term with C-section, reporting no complications.

The mean hospitalization duration was 1.5 ± 0.8 days, ranging from 1 to 4 days. Patients with no complications during the procedure were discharged home the same day, while those with limited hemorrhage after 48 h. The only patients requiring hysterectomy had an overall hospitalization of 96 h.

## 4. Discussion

The present study demonstrated the feasibility and effectiveness of local methotrexate injection followed by hysteroscopic resection to treat CSP with a low complication rate.

Our technique showed a higher success rate when compared with medical treatment alone (94.8% vs. 77%), with a short hospitalization (1.5 days) [17]. The proposed procedure also demonstrated good fertility preservation with 15.8% spontaneous pregnancy and term delivery, and the maintenance of a healthy uterus in nearly all patients.

Concerning the medical approach alone, systemic MTX administration has been investigated by three RCTs and several case series which showed a combined success rate of 75%, and a major complication rate of 13% [18]. In our study population, no complications were reported during or after local MTX injection. Instead, there were two cases of spontaneous expulsion and complete resolution of the CSPs.

Other more recently introduced methods such as uterine artery embolization or high-intensity focused ultrasound with or without methotrexate administration have been reported in small series with low complication and high success rates [18]. However, these methods are sophisticated, associated with postprocedural pain and not always available in every hospital [19,20].

Our study reported a success rate of 94.8%, with major complication in 5.2% of the patients, which is line with other studies reporting local injection or hysteroscopic treatment for CSP. Hysteroscopy treatment of CSP has been reported only in small series that include a total of 95. Of those, only 11 had preoperative systemic methotrexate, with only 3.2% experiencing major complications and 17% requiring additional treatment [17].

A recent retrospective study including 14 cases of CSP reported an enhanced effect of a direct injection of methotrexate in addition to one dose of systemic methotrexate pregnancy with a 7% failure rate [7].

Additionally, in 2019, Naeh et al. reported a series of 12 patients in whom the combined systemic and local methotrexate injection led to successful outcome in all cases without surgical intervention and or significant side-effects, but with a mean hospitalization of 9 days [9]. However, the mean gestational age was 7.5 weeks with no case exceeding 10 weeks, which might have impaired the efficacy of the treatment.

In the present study, the local injection of methotrexate followed by hysteroscopy likely increased the success rate facilitating the surgical removal of the persistent trophoblastic tissue. The ultrasonographic assessment of the perilesional blood was considered a factor for the correct timing of the procedure [5]. In our casuistic, weak blood flow at the first ultrasound scan was linked to a favorable prognosis and could be a marker of good outcome of the subsequent hysteroscopic procedure. However, both enhanced and strong vascularization were found in the patients with no complications, questioning the direct linkage between first ultrasound findings and the outcome. Nevertheless, the only serious complication occurred in a patient where the diagnosis was made at 11 weeks.

The remaining gestational tissue can be removed by hysteroscopy in combination with vascular coagulation of the implantation site. The use of hysteroscopy facilitates the achievement of a sufficient hemostasis with electrocauterization under visual control using a wire loop or rollerball, allowing the surgeon to have an extra tool to control the bleeding when compared to D&C.

However, the hysteroscopic treatment of CSP cannot be considered a routinary hysteroscopic procedure. It requires a high level of eye–hand coordination and is technically demanding, since meticulous coagulation of the implantation site is needed to prevent blockage of the operative field [21]. Laparoscopically assisted hysteroscopy for CSP is reported in the literature, allowing immediate identification and surgical repair of potential perforation of the uterus or the bladder [22,23]. Kresowik et al. suggested transabdominal ultrasound as a safe and low-cost aid for difficult hysteroscopic surgeries, especially in cases of synechiae and septa [24]. Sheng et al. used this minimally invasive technique to monitor intrauterine fluid in a case of cervical heterotopic pregnancy [25]. In our surgeries, transabdominal ultrasound guidance was used during dilation of the uterine cervix to avoid blind uterine perforation or damage to the CSP area with subsequent bleeding, thus making the hysteroscopic procedure difficult from the beginning.

The need for surgical removal of the persistent material is well reported in the literature. Recently, SMFM guidelines have suggested operative resection (transvaginal or laparoscopic), or ultrasound-guided vacuum aspiration of CSP as a first-line treatment [26]. However, surgical removal of CSP is associated with a high risk of complications. Amongst the investigated methods used to treat CSP, D&C is one of the most reported, but is associated with a complication rate standing between 21% (52% of which require additional treatment) and 62% [18,27].

In 2012, Timor-Trisch and Monteagudo demonstrated that of all treatment modalities for CSP, local Methotrexate had the lowest complication rate of 9.6% [27]. Surgical treatment alone achieved a success rate of 83% with 18% complication rate, compared to 7% for medical treatment according to other studies [17,18].

A recent UK national cohort study by Harb et al. reported a success rate of 96% and complication rate of 36% for surgical treatment [28]. Our combined technique with local MTX injection and hysteroscopic removal of CSP remnants achieved similar results, with one major complication such as uterine rupture with massive hemorrhage and subsequent hysterectomy (5.2%). Our results are also in line with the scarce literature available for combined medical and surgical treatment of CSP with D&C, whose success rate, hemorrhage rate and hysterectomy rate stand at 82%, 13% and 4%, respectively [18].

Limitations of our study are the small sample size and the absence of a control group, which reduce the generalizability of our study findings. Notably, the prevalence of the condition among the general population is low, thus making it difficult to achieve a wider study population with potential for comparison. Larger prospective comparative studies are needed to confirm the efficacy and safety of MTX injection plus hysteroscopic resection for CSP treatment.

## 5. Conclusions

Several methods have been reported but the quality of the studies is low and they are mainly case series that do not allow a conclusion. However, a combination of medical and surgical options seems to be the best way to achieve CSP removal and obtain a low risk of complications.

The combination of local methotrexate injection followed by hysteroscopic resection of the residual pregnancy tissue seems to be a valuable therapeutic option for CSP, with a high success rate, low risk of major complications and potential for fertility preservation. In this instance, the perisaccular blood flow evaluation could be a marker of final success and could be routinely proposed to establish the time of surgery. In addition, in high-risk patients with persistent enhanced perisaccular blood flow, bleeding-diminishing procedures such as Foley balloons should be considered at the end of the procedure.

Despite the apparent feasibility of this combined technique, further larger prospective comparative studies are needed to confirm the efficacy of this procedure.

## Figures and Tables

**Table 1:** Clinical characteristics, procedure details and outcomes of the study population.

Patient	Age at Diagnosis	Previous C-Section (*n*)	TVUS Perisaccular Flow Sign at Diagnosis	Gestational Age * at MTX Injection	Time between MTX Injection and Hysteroscopic Resection (Weeks, *n*)	Surgical Complications	Blood Transfusion	Final Status
1	28	2	Weak	7 + 4	4 weeks	None	None	Healthy uterus
2	27	3	Weak	8 + 1	6 weeks	None	None	Healthy uterus
3	34	5	Enhanced	9 + 1	8 weeks	Hemorrhage	1 unit PRBC	Healthy uterus
4	36	5	Weak	7 + 3	4 weeks	None	None	Healthy uterus
5	28	1	Weak	8 + 2	4 weeks	None	None	Healthy uterus, pregnancy
6	33	3	Weak	9 + 0	Spontaneous expulsion	None	None	Healthy uterus
7	30	2	Strong	11 + 5	12 weeks	Hemorrhage, uterine rupture	1 unit PRBC	Hysterectomy
8	31	3	Weak	8 + 4	8 weeks	None	None	Healthy uterus
9	29	1	Enhanced	6 + 1	4 weeks	None	None	Healthy uterus, pregnancy
10	37	5	Enhanced	8 + 0	6 weeks	Hemorrhage	None	Healthy uterus
11	29	2	Enhanced	7 + 6	4 weeks	None	None	Healthy uterus
12	31	3	Enhanced	9 + 6	8 weeks	None	None	Healthy uterus
13	26	1	Weak	7 + 2	3 weeks	None	None	Healthy uterus, pregnancy
14	28	2	Enhanced	7 + 3	4 weeks	None	None	Healthy uterus
15	35	2	Weak	6 + 3	1 week	None	None	Healthy uterus
16	34	5	Weak	6 + 6	Spontaneous expulsion	None	None	Healthy uterus
17	28	2	Enhanced	8 + 4	4 weeks	Hemorrhage	None	Healthy uterus
18	32	2	Strong	8 + 2	8 weeks	Hemorrhage	1 unit PRBC	Healthy uterus
19	24	2	Enhanced	7 + 3	4 weeks	Hemorrhage	None	Healthy uterus
20	28	3	Strong	8 + 1	3 weeks	Hemorrhage	None	Healthy uterus
21	29	2	Strong	7 + 3	3 weeks	None	None	Healthy uterus

* expressed in weeks plus days of gestation. Abbreviations: C-section, caesarean section; *n*, number; MTX, methotrexate; PRBC, packed red blood cells.

## Data Availability

The data presented in this study are available on request from the corresponding author. The data are not publicly available due to the need to maintain the privacy of patients.
